# School Incivility and Academic Burnout: The Mediating Role of Perceived Peer Support and the Moderating Role of Future Academic Self-Salience

**DOI:** 10.3389/fpsyg.2019.03016

**Published:** 2020-01-28

**Authors:** Qiyu Bai, Shuang Liu, Tomoko Kishimoto

**Affiliations:** ^1^The School of Journalism and Communication, Renmin University of China, Beijing, China; ^2^Research Center of Journalism and Social Development, Renmin University of China, Beijing, China; ^3^Department of Social Psychology, Zhou Enlai School of Government, Nankai University, Tianjin, China

**Keywords:** school incivility, academic burnout, perceived peer support, future academic self-salience, moderated mediation model

## Abstract

This study examined a mediation model about whether perceived peer support (PPS) mediates the link between school incivility and academic burnout. More importantly, we also investigated how future academic self-salience (FASS) as a trait moderates this mediated relationship. We collected data from a sample of 475 students by a two-wave survey. Results indicate that PPS mediated the relationships for school incivility with academic burnout. Moderated mediation analysis intended to further reveal that PPS mediated the relationship for only those students with high FASS while what the current findings found are the separate effects of the mediation of PPS on the relationship between school incivility and academic burnout and the moderation of FASS on the relationship between PPS and academic burnout. Therefore, the findings underscore the significance of influence from peer relationships when investigating the relationship between school incivility and academic burnout. Further evidences are needed to prove the mediated moderation role of FASS.

## Introduction

Burnout was first defined by Maslach and Jackson (1981) to refer to a syndrome of emotional exhaustion, cynicism, or depersonalization and lack of personal accomplishment. Burnout was assumed to occur in individuals in many occupations, for example, in health care, social service, or education (Schaufeli et al., 2002b). Burnout is experienced by students as well (Schaufeli et al., 2002a; Salmela-Aro et al., 2008), which is called academic burnout (Rios-Risquez et al., 2018; Wang et al., 2018). Symptoms of student burnout are also supposed to have those three dimensions: emotional exhaustion, depersonalization, and lack of personal accomplishment (Schaufeli et al., 2002b). When academic burnout happens, both their academic performance and well-being can be affected. On the one hand, studies have shown that burnout negatively correlates with dropout and diminished academic and cognitive performance (Bask and Salmela-Aro, 2013; May et al., 2015; Palos et al., 2019). On the other hand, burnout consistently influences students’ adjustment to school life and psychological well-being, which may eventually lead to depression and suicide (Fiorilli et al., 2017; Rios-Risquez et al., 2018).

In the last two decades, incivility has been researched intensively in the organizational behavior literature, namely, workplace incivility (Andersson and Pearson, 1999; Schilpzand et al., 2016). Recently, the concept has been applied to the family context (Lim and Tai, 2014). However, it has not been attached with much importance in the school context. We then suggest the concept of school incivility. It is a kind of deviant behaviors occurring in students’ interactions with each other, which is low-intensity but has an ambiguous intention that violates the norms of mutual respect among students. Similar as incivility in other domains (Lim and Tai, 2014), school incivility can be concluded with three characteristics: (a) it is less intense than school bullying or violence; (b) it has ambiguous purposes to harm; (c) it violates the norms of mutual respect between classmates or peers. As research in workplace incivility has already shown, incivility is costly and pervasive, and it has significant negative consequences on affection, cognition, and behaviors not only for its targets but also for its witnesses and instigators (Schilpzand et al., 2016). We thus speculate that school incivility may have similar impacts on students. However, its impact on the degree of academic burnout has not been investigated yet. In the current study, we thus consider, for adolescents in the school context, incivility constructs a relatively harmful environment for students, then it is supposed to be one of the important reasons for academic burnout. Then, we also intended to find the mechanism of the relationship.

As one of the major sources of social support during adolescence, peer support is a fundamental part of adolescents’ social and psychological environment (Song et al., 2015). According to the conservation of resources (COR) theory, one of the leading theories in explaining stress and burnout (Gorgievski et al., 2011), environmental circumstances often threaten or cause a depletion of people’s resources, and once resources are threatened, stress could result. Given that peer support is a kind of resource people need, we suppose that it is a mediator between school incivility and academic burnout. Researches about workplace incivility have shown that incivility from various social relationships is negatively related to perceived social support (Lim and Lee, 2011). Thus, we suppose that adolescents who experience more incivility are inclined to have lower perceived peer support (PPS). Meanwhile, research has repeatedly found that perceived much social support relates negatively to academic burnout (Kim et al., 2018), and Yang (2004) found that social support has a negative direct effect on burnout. Thus, we consider that PPS is negatively associated with academic burnout. Considering the COR theory and these evidences, therefore, we hypothesize that PPS mediates the relationship between school incivility and academic burnout.

Moreover, building on the COR theory, we suppose that after resources are threatened, different people may act out a different degree of the outcome. Ashforth (2000) suggested that differences in the salience of identities could allow individuals behave differently in the same social context. Future academic self-salience (FASS) is adapted from the concept of future work self-salience (FWSS), which is the salience of future self in relation to study. Originally, FWSS refers to individuals’ representation of themselves in the future that reflects their hopes and aspirations related to work (Strauss et al., 2012). Here, we define FASS as the degree to which individuals are motivated to prepare for study and whether future self about academic achievement is clear and lucid for a person. Theoretically, it is believed that future self-salience, which evolved from “possible selves,” can facilitate the achievements of individuals’ future development (Markus and Nurius, 1986). Former research about FWSS has found that it increases the likelihood of proactive career behaviors and career adaptability (Guan et al., 2014; Cai et al., 2015; Taber and Blankemeyer, 2015) and has focused on the positive effects brought by future self-salience (Strauss et al., 2012). However, potential downsides of FASS have yet been researched much. Yu et al. (2016) have investigated the adverse effect of high FWSS. They found that the indirect effect of abusive supervision on sales performance *via* affective commitment was stronger for employees with higher FWSS. According to Yu et al. (2016), its characteristic of future-oriented also enables individuals to realize the discrepancy between future and reality and be more aware and sensitive to the situation constraints (Atance and O’Neill, 2001; Yu et al., 2016). Therefore, in the present study, we suppose that adolescents with high FASS would be more easily affected by decreased peer support and then present psychological outcomes, that is, academic burnout. In other words, we expected that higher levels of FASS would amplify the detrimental effect of PPS on academic burnout. In contrast, for adolescents with low FASS, due to their lack of aim on study and ambiguous future selves, no matter how peer support is, they will not be stimulated too much to cope with their study so that the likelihood of academic burnout is less than that of adolescents with high FASS.

**FIGURE 1:**
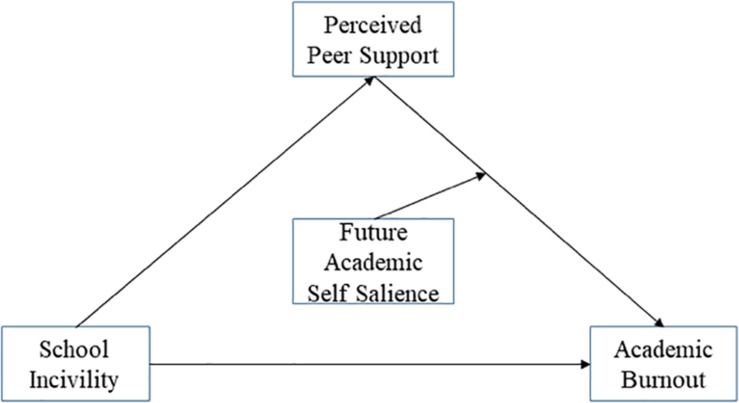
Proposed moderated mediation model.

Meanwhile, as aforementioned, PPS was expected to mediate the relationship between school incivility and academic burnout. We anticipated that FASS will moderate the second half of the mediation model, that is, the relationship between PPS and academic burnout rather than others. Specifically, both school incivility and peer support are in relation to interpersonal relationship and have closer associations with each other. Therefore, we put forward that FASS is more likely to have the interaction effect with PPS rather than school incivility, and we expected that FASS will moderate the indirect effect of school incivility on academic burnout through PPS (a moderated mediation model), as depicted in Figure 1. The mediation effect of PPS on the relationship between school incivility and academic burnout will be stronger for adolescents who have higher salience of their future academic selves, presenting the adverse effect of FASS.

To conclude, the current study examined the moderated mediation among incivility, peer support, FASS, and academic burnout. Specifically, we hypothesized that (as Figure 1 shows): (1) school incivility is positively related to academic burnout; (2) PPS is negatively related to school incivility and academic burnout; (3) PPS mediates the relationship between school incivility and academic burnout; (4) FASS strengthens the mediated relationship between school incivility and academic burnout through PPS, that is, the path between PPS and academic burnout is stronger when FASS is high.

## Materials and Methods

### Participants and Procedure

Data were collected from high school students in Hebei Province, China, by paper-and-pencil surveys two times. We randomly assigned each participant a subject ID number in advance. Then, participants are asked to answer the surveys with their ID number. At Time 1, a total of 475 students attended, and they have assessed levels of school incivility and submitted their demographics information (age and gender). After 1 month, at Time 2, participants’ PPS, FASS, and academic burnout were measured. Among the former 475 students of Time 1, 418 participants completed the second survey (88.0% response rate). By matching ID numbers, we acquired the whole two-wave data for the present study. The final sample consisted of 219 females and 199 males, ranging from 14 to 19 years old.

### Measures

#### School Incivility

School incivility was measured by an adaptive version of the Family Incivility Scale (Lim and Tai, 2014) and Workplace Incivility Scale (Cortina et al., 2001). It includes six items that are parallel to those of the original Family Incivility Scale, assessing the frequencies of incivility students experienced when in interactions with their classmates in the past month like “How often do your classmates put you down or condescending to you?” or “How often do your classmates pay little attention to your statement or show little interest in your opinion?” The instruction is translated and adjusted for the research targeted sample. Participants responded to the six items on a five-point scale, which asked them if any of their peers engaged in behaviors violating mutual respect. Cronbach’s alpha for the scale was 0.742.

#### Perceived Peer Support

Perceived peer support was assessed by the Song et al. (2015) measurement of perceived support from peers. It assesses students’ perceptions of peer support and positive relationships among peers. These four items are “Students get along well in my school,” “It is easy to make friends in my school,” “My peers at school respect and care about me,” and “I can solicit my peers’ help when performing difficult tasks.” Except possibly for the last statement, these items generally describe emotional support from peers. Participants respond to them on a seven-point scale. They formed a single peer support factor in exploratory factor analysis (Song et al., 2015). Cronbach’s alpha for the scale was 0.898.

#### Future Academic Self-Salience

Future academic self-salience was measured with a modified Chinese version of FWSS Scale (Strauss et al., 2012; Guan et al., 2014; Cai et al., 2015). It assesses the degree to which future selves about academic achievement is clear and lucid for individuals. It includes five items on a five-point scale. These items are “I can easily imagine my future academic self”; “The mental picture of this future is very clear”; “This future of academic self is very easy for me to imagine”; “I am very clear about who and what I want to become in my future academic”; and “What type of future I want in relation to my academic is very clear in my mind.” The Cronbach’s alpha of the scale was 0.853.

#### Academic Burnout

Academic burnout was measured with the Chinese version of Maslach Burnout Inventory–Student Survey (MBI-SS) (Schaufeli et al., 2002a). It is a seven-point scale, with 15 items assessing exhaustion, cynicism, and efficacy. The present study only used the total scores of all items. Higher scores reflect a higher degree of academic burnout. Cronbach’s alpha for the total scale was 0.931.

### Data Analysis

The major goal of the present study is to examine a moderated mediation hypothesis. The present study hypothesized the mediating relation of school incivility → PPS → academic burnout and that FASS moderated the relationship between the mediator and academic burnout. First, we present the result of descriptive analysis and bivariate correlation analysis. Then we transformed variables into Z-score for hierarchical multiple regressions by SPSS PROCESS macro suggested by Hayes (2012) to test the hypotheses.

## Results

### Descriptive Analysis and Bivariate Correlations Analysis

Results of descriptive statistics, bivariate correlations, and Cronbach’s alphas of all variables are in Table 1. Consistent with former research, school incivility was significantly correlated with academic burnout (*r* = 0.28, *p* < 0.01). The more school incivility one experiences, the more likely one will be to present a high level of academic burnout. Also, PPS was in negative correlation with school incivility (*r* = −0.15, *p* < 0.01) and academic burnout (*r* = −0.17, *p* < 0.01). Moreover, demographic variables (i.e., gender and age) were significantly correlated with academic burnout. Therefore, in all later analyses, we set gender and age as covariates and included them in further analysis.

**TABLE 1:** Descriptive statistics and bivariate correlations.

Variable	*M*	*SD*	1	2	3	4	5	6
(1) Gender	1.52	0.50	\					
(2) Age	16.11	0.76	−0.28**	\				
(3) School incivility	2.97	1.74	–0.02	0.04	(0.74)			
(4) PPS	4.11	0.63	0.07	–0.01	−0.15**	(0.90)		
(5) Academic burnout	2.86	1.34	−0.19**	0.21**	0.28**	−0.17**	(0.93)	
(6) FASS	4.31	1.21	0.04	–0.01	–0.05	0.26**	−0.10*	(0.85)

Figures in parentheses are Cronbach’s alphas. PPS, perceived peer support; FASS, future academic self-salience. *p < 0.05; **p < 0.01.

### Predicting Changes in Academic Burnout

First, the total direct effect of school incivility on academic burnout was tested. As Table 2 showed, after controlling for gender and age, school incivility was negatively related to PPS (β = −0.13, *p* < 0.05). Next, the mediation effect without the moderator was tested. After PPS was taken into account, the effect of school incivility was still significant while *R*^2^ changed from 0.02 to 0.10 and *p*-value also became more salient (*p* < 0.001), which suggests partial mediation. Then, indirect effects were analyzed. Results showed that the bootstrapped confidence interval (CI) did not include zero (95% CI: 0.01, 0.06), and the indirect effect of school incivility on academic burnout through PPS is significant (effect = 0.02). Therefore, the intervening effect of PPS for school incivility was significant. Taken together, it is supported that PPS mediates the relationship between school incivility and academic burnout. Finally, we examined whether the interaction for PPS with FASS was significant in predicting academic burnout. Results presented in Table 2 showed that FASS interacted with PPS in predicting academic burnout significantly. However, the moderated mediation effect was insignificant (95% CI: −0.01, 0.03), indicating that the indirect effect of school incivility on academic burnout through PPS was not significantly moderated by FASS. Therefore, our hypothesis of the moderated mediation relationship was not totally supported.

**TABLE 2:** Results for testing hypotheses.

	*B*	*SE*	*t*	LLCI	ULCI	*R*^2^	*F*
**Mediation analysis**							
Outcome variable: PPS							
Constant	–0.71	1.16	–0.61	–2.99	1.58	0.02	2.70*
Gender	0.11	0.10	1.08	–0.09	0.32		
Age	0.03	0.07	0.48	–0.10	0.17		
School incivility	–0.13	0.05	−2.54*	–0.23	–0.03		
Outcome variable: academic burnout							
Constant	–0.99	1.49	–0.66	–3.91	1.94	0.10	11.03***
Gender	–0.31	0.13	−2.36*	–0.58	–0.05		
Age	0.27	0.09	3.05**	0.10	0.44		
School incivility	0.18	0.06	2.82**	0.06	0.31		
PPS	–0.18	0.06	−2.85**	–0.31	–0.06		
**Moderated mediation analysis (moderation effect of M-Y)**							
Outcome variable: PPS							
Constant	–0.69	1.17	–0.59	–3.00	1.61	0.02	2.47
Gender	0.10	0.11	0.95	–0.11	0.31		
Age	0.03	0.07	0.49	–0.10	0.17		
School incivility	–0.12	0.05	−2.47*	–0.22	–0.03		
Outcome variable: academic burnout							
Constant	–0.78	1.49	–0.52	–3.72	2.15	0.11	8.10***
Gender	–0.35	0.13	–2.59	–0.61	–0.08		
Age	0.26	0.09	2.94	0.09	0.43		
School incivility	0.18	0.06	2.78**	0.05	0.31		
PPS	–0.18	0.07	−2.74**	–0.31	–0.05		
FASS	–0.06	0.07	–0.81	–0.19	0.08		
PPS × FASS	–0.10	0.05	−2.02*	–0.20	–0.01		

PPS, perceived peer support; FASS, future academic self-salience; LL, low limit; CI, confidence interval; UL, upper limit. Gender was dummy-coded. Gender and age are set as covariates. Bootstrap sample size = 5,000. *p < 0.05; **p < 0.01; ***p < 0.001.

**FIGURE 2:**
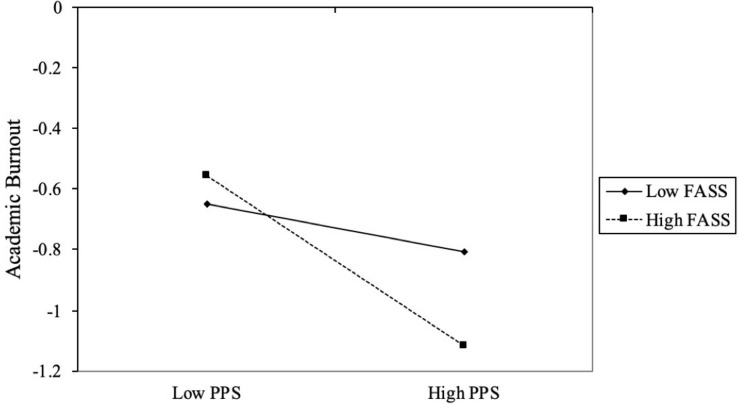
Interaction of PPS and academic burnout on FASS. PPS, perceived peer support; FASS, future academic self-salience. High and low levels of PPS and academic burnout represent one standard deviation above and below the mean, respectively.

We also operationalized high and low levels of FASS as one standard deviation above and below the mean score as Preacher et al. (2007) suggested to examine whether and how PPS is different for adolescents across high and low levels of FASS. Results indicated that the degree of PPS was stronger and significant in the high FASS condition (*b*_*high FASS*_ = −0.28, *p* < 0.001) but was weaker and not significant in the low FASS condition (*b*_*low FASS*_ = −0.08, *p* > 0.05) (Table 3). Predicted PPS against academic burnout separately for low and high levels of FASS (1 *SD* below the mean and 1 *SD* above the mean, respectively) was plotted (Figure 2). Therefore, it is supported that higher levels of FASS would amplify the effect of PPS on academic burnout.

**TABLE 3:** Results of the simple slopes analysis for PPS to academic burnout.

Moderator		PPS		
	Level	Gradient	*t*	*p*
FASS	Low	−0.08	−0.98	0.33
	High	−0.28	−0.37	0.00

*FASS, future academic self-salience; PPS, perceived peer support.*

To conclude, FASS does moderate the relationship between PPS and academic burnout while it failed to moderate the mediated relationship between school incivility and academic burnout *via* PPS.

## Discussion

Nowadays, adolescents from various countries and regions have problems with their studies and school life. Among them, school-related problems considering social relationships and academic burnout are of great importance. Although recent studies have devoted much attention to workplace incivility and extended the concept to the family context, little research has investigated the phenomenon of incivility happening in the school context. However, although less intense than school bullying or violence, the influence of school incivility cannot be ignored due to its ambiguous purposes to harm and create a negative impact on the peer relationship as well as the school environment. The present results show that having experienced school incivility, adolescents are likely to present a higher degree of academic burnout. The findings suggest that school incivility can create stress and contribute to negative psychological and academic outcomes. This is consistent with former research, which has clarified that incivility in the workplace is of high prevalence and high costs (Schilpzand et al., 2016) and that incivility in the family also has negative consequences at work (Lim and Tai, 2014). Therefore, it is of great importance to improve the school climate to keep school incivility at bay, for example, to raise awareness among students about school incivility and enhance anti-incivility norms and ability (Menesini and Salmivalli, 2017). Besides, results show that PPS mediates the impact of school incivility on academic burnout after controlling for age and gender. Consistent with former research on negative outcomes of incivility in workplace and family context (Andersson and Pearson, 1999; Cortina et al., 2001; Lim and Tai, 2014; Bai et al., 2016; Schilpzand et al., 2016), our study also reveals that exposure to school incivility is negatively associated with PPS and positively associated with academic burnout. PPS associated with experiencing school incivility appears to be a crucial reason for academic burnout.

However, our proposed moderated mediation model is partly supported. Adapted from FWSS (Strauss et al., 2012), the concept of FASS refers to individuals’ hopes and aspirations in relation to study. It is also a kind of identity-based motivation (Taber and Blankemeyer, 2015), which facilitates future-oriented activities and decision makings (Lin et al., 2016; Leech et al., 2019; McCue et al., 2019). It also implies an individual’s flexibility or willingness to make changes to oneself or environments to achieve adaptive outcomes (Guan et al., 2017). Originally, we supposed that under the influence of school incivility, FASS may have the buffering effect for the impact of the mediator PPS on academic burnout. However, although FASS does moderate the relationship between PPS and academic burnout, the indirect effect of school incivility on academic burnout *via* PPS could not be significantly influenced by FASS. Meanwhile, although FASS fails to be an important indicator of academic burnout when PPS is affected by school incivility in the present study, our results may still suggest that FASS has an important role in the second half of the path and indicate the potential adverse effect of high FASS, which is consistent with former research (Yu et al., 2016). Therefore, for ameliorating students’ academic burnout, it is crucial to recognize the salience of their future academic selves and keep providing them with peer support at the same time. To deepen our understanding of the moderating effects of FASS on the relationship, both theoretical research and empirical research are needed.

Several limitations need to be discussed. First, the causal relationship—school incivility leads to decreased peer support and then contributes to academic burnout—cannot be definitely proven in our present research design. Future research could manipulate incivility in an experimental context and adopt a longitudinal research design, investigating detailed information about the influence of peer relationships as well as students’ self-perceptions about peer support. Second, the present study fails to prove the hypothesized moderated mediation model. While the mediation relationship is supported, the moderated mediation model is not supported as we anticipated. Therefore, future research is needed to investigate the role of FASS. Third, due to our consideration on correlation relationship and for reducing common method bias, we have not tested PPS and academic burnout in Time 1, although the findings already meet our anticipation for the specific relationships. Furthermore, our model is established on perceptual measures, and all variables are assessed with self-ratings. Future studies can search for explicit evidences, such as academic performance, team cooperation, or later career success. Finally, as our study was conducted in China, it may not be generalized to other cultures. Future research could explore cross-culture differences.

Notwithstanding the limitations, the findings of this study may extend our knowledge in terms of explaining the mechanism of academic burnout and clarifying the relationship between school incivility, PPS, FASS, and academic burnout. Although our moderated mediation model is not fully supported in that FASS fails to buffer the negative outcomes of the mediation of PPS in the relationship in school incivility’s impacts on academic burnout, it moderates the relationship between PPS and academic burnout. The findings might suggest a new perspective on school counseling interventions for academic burnout and give much concern to school practitioners and administrators.

## Data Availability Statement

The datasets generated for this study are available on request to the corresponding author.

## Ethics Statement

The studies involving human participants were reviewed and approved by the Renmin University of China. Written informed consent to participate in this study was provided by the participants’ legal guardian/next of kin.

## Author Contributions

QB planned and conducted this study. SL conducted the data analysis and initiated the draft of the article. TK supervised the study and revised the article.

## Conflict of Interest

The authors declare that the research was conducted in the absence of any commercial or financial relationships that could be construed as a potential conflict of interest.
